# Supplementary material for: The interactome of intact mitochondria by cross-linking mass spectrometry provides evidence for coexisting respiratory supercomplexes
Source: Mol Cell Proteomics. 2017 Dec 8;17(2):216–32. doi: 10.1074/mcp.RA117.000470 (PMC5795388; doi:10.1074/mcp.RA117.000470)
Supplement: Supplemental Data [file supp_RA117.000470_133922_0_supp_23978_fzffwf.zip › spectra_annotation/mito_DR_spectra_annotation/338-1-5-1-2-1.pdf]

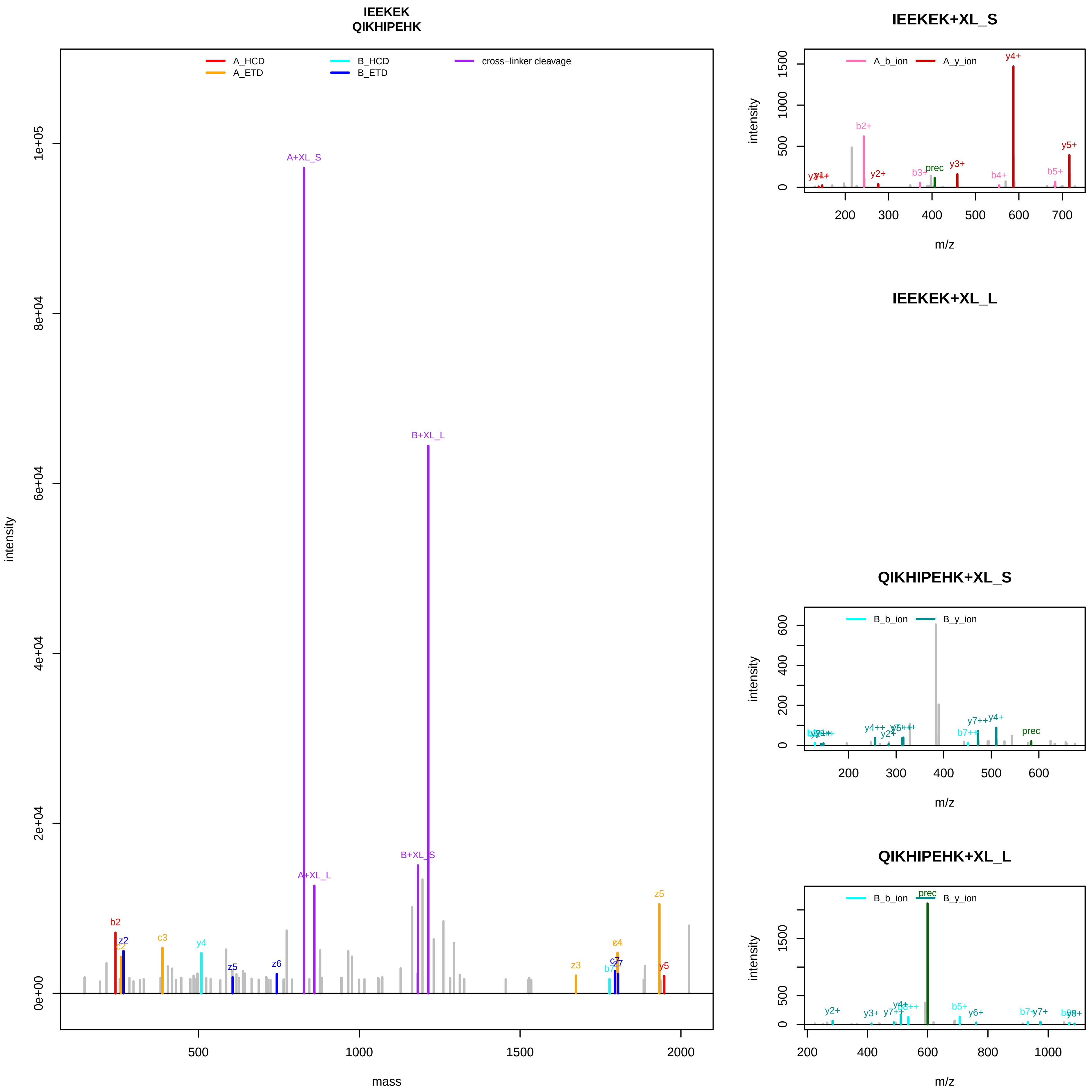

IEEKEK
QIKHIPEHK
A_HCD
A_ETD
B_HCD
B_ETD
cross−linker cleavage
A+XL_S
B+XL_L
B+XL_S
A+XL_L
b2
z2
c2
c3
y4
z5
z6
b7
c7
z7
z4
z3
z5
y5
intensity
mass
0e+00
2e+04
4e+04
6e+04
8e+04
1e+05
500
1000
1500
2000
IEEKEK+XL_S
A_b_ion
A_y_ion
y4+
b2+
y5+
prec
y3+
y2+
b3+
b4+
b5+
intensity
m/z
IEEKEK+XL_L
QIKHIPEHK+XL_S
B_b_ion
B_y_ion
y7++
y4+
b7++
y2+
y4++
prec
intensity
m/z
QIKHIPEHK+XL_L
B_b_ion
B_y_ion
prec
y2+
y3+
y4+
b5+
y6+
y7+
intensity
m/z
